# Osseous Metaplasia of the Endometrium: A Rare Pathological Entity

**DOI:** 10.7759/cureus.62204

**Published:** 2024-06-11

**Authors:** Sarita Devdhar, Vertika Gupta, Rashmi Gautam, Arun Chaudhary

**Affiliations:** 1 Pathology, Noida International Institute of Medical Sciences, Greater Noida, IND; 2 Pathology, Saraswathi Institute of Medical Sciences, Hapur, IND

**Keywords:** histopathological examination, abnormal uterine bleeding, infertility, endometrium, osseous metaplasia

## Abstract

Osseous metaplasia of the endometrium is a rare and intriguing pathological condition characterized by the presence of bony tissue within the endometrial cavity. This phenomenon can have significant clinical implications, particularly in the context of infertility. The etiology of osseous metaplasia remains unclear, although various hypotheses have been proposed, including chronic inflammation, dystrophic calcification, and residual embryonic tissue. Clinically, patients may present with secondary infertility, abnormal uterine bleeding, or pelvic pain. Diagnosis can be made based on ultrasonography and histopathological analysis of the endometrial tissue. Treatment typically involves the removal of the osseous tissue via hysteroscopy, which can lead to the restoration of normal endometrial function and potentially resolve infertility. Further research is needed to elucidate the etiological factors and optimize treatment protocols.

## Introduction

Endometrial osseous metaplasia is a rare clinical entity, with very few cases reported both in India and worldwide [1-6]. It is characterized by the presence of immature or mature bone in the endometrium [2-6]. Its pathogenesis is not clear, but the most widely accepted theory is metaplasia of the stromal cells into osteoblasts, which produce osseous tissue [2,3,7]. Most of the patients belong to the reproductive age group [3,4,8] and presented with infertility with a prior history of either therapeutic or spontaneous first-trimester abortion [3,4,6,8-10]. The presence of bone in the endometrium can be confirmed through ultrasound, hysteroscopy, or histopathological examination (HPE) following a biopsy or curettage. Management includes hysteroscopic evacuation of these bony spicules [7,8,10] and most of the patients are able to conceive after the evacuation [2,4,6,8-10].

The authors hereby describe a case of endometrial osseous metaplasia in a 38-year-old female who presented with complaints of polymenorrhagia.

## Case presentation

We present the case of a 38-year-old female patient who was admitted to the gynecology ward with the chief complaint of polymenorrhagia for the past year. She had no other relevant past or present medical history, and her last live birth was 10 years ago. The patient was afebrile, and her vitals were within normal limits. A bimanual pelvic examination revealed an eight- to 10-week-size uterus with a healthy cervix. A complete blood count showed mild anemia, with hemoglobin at 10.2 g/dL. All other routine investigations were within normal limits. Ultrasound findings included areas of calcification in the uterus. The patient was started on progesterone therapy, and an endometrial biopsy was performed and sent for HPE.

A gross examination of the biopsy revealed multiple gray-brown tissue pieces, measuring 2.5 × 2.0 × 0.5 cm in total. H&E-stained sections showed irregularly spaced endometrial glands embedded in loose stroma and surrounded by surface endometrium. Some glands were in the proliferative phase, while others showed early secretory changes. In certain areas, gland overcrowding and angulated glands were observed. The stroma exhibited a dense inflammatory infiltrate, primarily composed of neutrophils, along with a few plasma cells and lymphocytes. Numerous fragments of mature and immature bony tissue were intermixed with endometrial fragments. Additionally, there were areas of hemorrhage and fibrin collection. Based on these microscopic features, the diagnosis was disordered proliferative endometrium with acute and chronic endometritis and osseous metaplasia.

**Figure 1:**
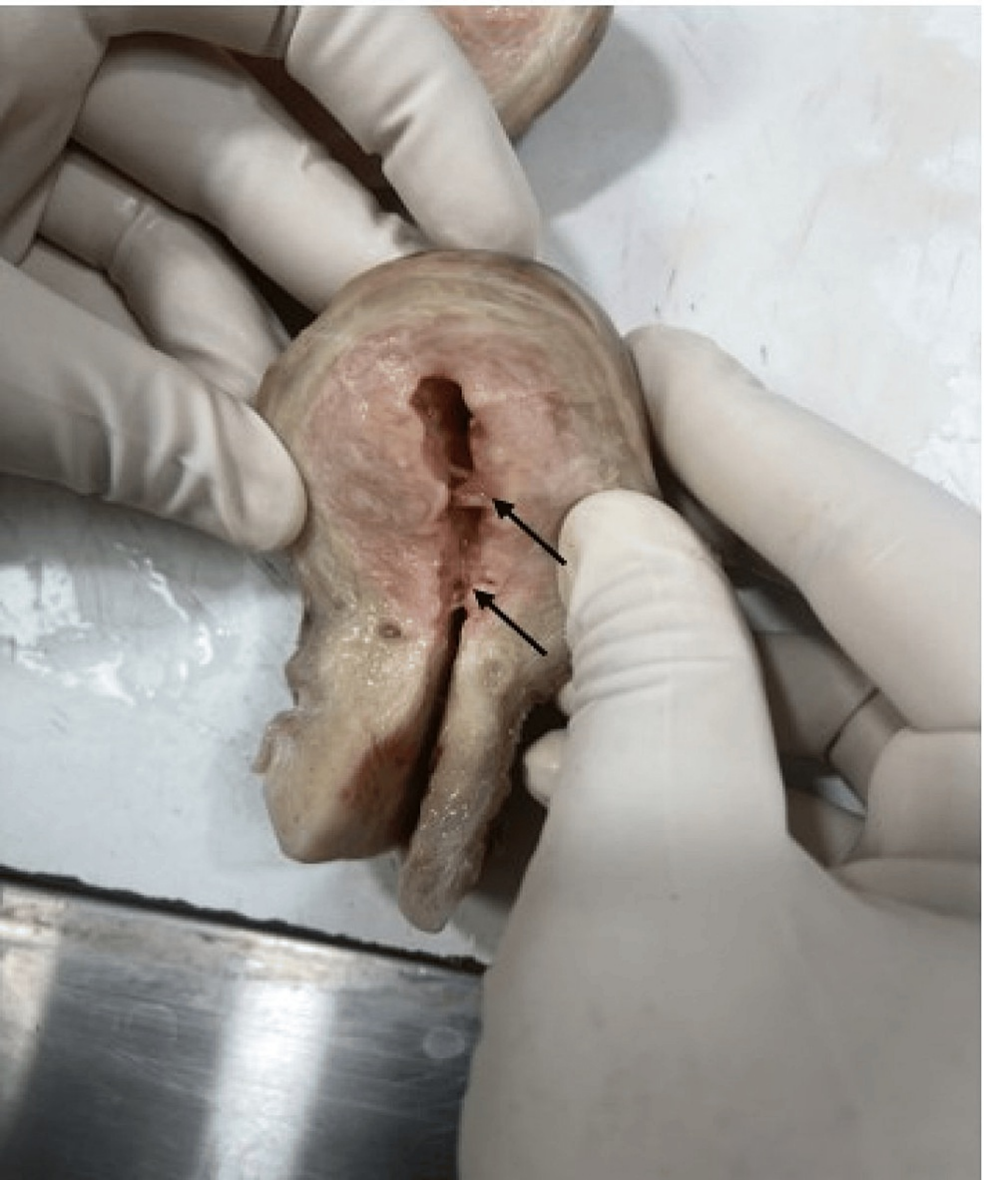
Gross specimen of the uterus with cervix showing a dilated uterine cavity with adhesions (marked with black arrows)

As the patient did not respond to hormonal and other interventions, a hysterectomy was planned. The specimen was sent for HPE. On gross examination, the uterus and cervix measured 10.0 × 6.0 × 3.5 cm. The external surface was unremarkable. The endometrial cavity was dilated and showed adhesions. The endomyometrium measured 2.2 cm at its thickest. A small cyst measuring 0.2 cm and filled with mucoid material was seen in the myometrium. The cervix measured 3.5 cm and appeared hypertrophied (Figure 1).

**Figure 2:**
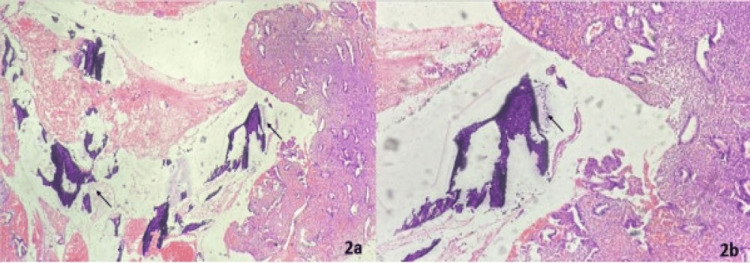
Photomicrographs showing endometrial fragments intermixed with mature bony tissue (marked with black arrow). The endometrium shows features of disordered proliferation with chronic endometritis (A: H&E stain, 40X; B: H&E stain, 100X)

On microscopic examination, the endometrium showed features of disordered proliferation with chronic endometritis. Fibrous and collagen bundles were observed in the stroma. Two foci of osseous metaplasia with calcification and osteoclast-type giant cells were also noted (Figure 2a, 2b). The myometrium exhibited deeply penetrating endometrial glands surrounded by endometrial stroma, consistent with adenomyosis. Chronic inflammatory infiltrates, comprising lymphocytes and plasma cells, were also observed in these foci. No foci of osseous metaplasia were found in the myometrium even after extensive grossing. The cervix displayed features of chronic cervicitis with squamous metaplasia. The postoperative course was uneventful, and the patient was discharged.

## Discussion

Endometrial stromal metaplasia is characterized by the presence of islands of smooth muscle, cartilage, and bone within the endometrial stroma. Osseous metaplasia of the endometrium is an unusual and rare pathological phenomenon, with only a handful of cases reported in the literature [1-6]. Garzon et al. [8] reported an incidence of 0.015% in women who underwent hysteroscopy. Despite its rarity, it poses diagnostic and therapeutic challenges for clinicians. It is characterized by the formation of immature or mature bone within the endometrial tissue [2-6].

It is more commonly seen in females in the reproductive age group [3,4,8], although a few cases have been described in postmenopausal females as well [5,11]. A history of previous abortions is seen in a large number of patients [3,4,6,8-10,12]. A time gap varying from eight weeks to 14 years may be seen between abortion and the development of osseous metaplasia in females of reproductive age [3,5,7,10,13]. The presence of bony tissue in the endometrium can result in a spectrum of symptoms, including infertility (the most frequent), abnormal uterine bleeding, and pelvic pain [2,3,6,8,9,11,12]. Endometrial bone can act as a foreign body, lead to menstrual irregularities, and serve as an intrauterine contraceptive device. Removal of the bone has been associated with the restoration of fertility and improvement in menstrual symptoms [2,4,6,8-10]. The index case belonged to the reproductive age group, and her chief complaint was polymenorrhagia. In contrast to findings by other authors, no history of antecedent abortion or problems in conception was seen in our case.

The exact etiology of endometrial osseous metaplasia remains unclear, yet several theories have emerged to elucidate the occurrence of osseous metaplasia. These include concepts such as heterotopia, retained fetal bones post-abortion, dystrophic calcification within retained products of conception, metabolic disorders leading to metastatic calcification, metaplasia in the course of tissue healing, and prolonged estrogen therapy [1,2,4,6,9,13]. One of the most widely accepted theories includes the metaplasia of multipotent stromal cells of the endometrium into osteoblasts that produce bony tissue [2,3,7]. In a study by Cayuela et al., where they examined DNA patterns, it was observed that various cell types, like pluripotent mesenchymal cells, fibroblasts, and Müllerian cells, undergo osseous metaplasia in response to inflammation and curettage [7]. Various authors [2,3,13] observed that endometrial mesenchymal cells can differentiate into chondroblasts or osteoblasts in the setting of chronic endometritis. The endometrial biopsy findings in our patient showed features of nonspecific chronic inflammation, suggesting a potential connection to the development of osseous metaplasia. Sugino et al. suggested that the functionality of the superoxide radical superoxide dismutase system, crucial for endometrial differentiation, could potentially extend to osseous metaplasia [14]. Prolonged inflammation after abortion, caused by retained products of conception, might stimulate the release of superoxide radicals or tumor necrosis factor from mononuclear phagocytes. A lack of protective superoxide dismutase activity in the endometrium could lead to the transformation of multipotent stromal cells into osteoblasts [14].

Pathologists must identify the nonneoplastic nature of osseous metaplasia to prevent misdiagnosing it as a malignant Müllerian tumor of the uterus [2,6,10,11]. It is essential to exclude the possibility of endometrial tuberculosis, considering tuberculosis ranks among the primary causes of infertility. The chronic inflammation observed in tuberculosis could potentially facilitate secondary osteogenesis [5]. Intrauterine retention of fetal bones following spontaneous abortions has been reported in the literature [15]. Rosa-e-Silva et al. observed that these cases share commonalities in terms of histories and symptoms [1]. The lack of surrounding tissue reaction and the presence of endochondral ossification may serve as distinguishing factors between osseous metaplasia and retained fetal tissue. Also, metaplasia is characterized by endogenous bone development, which is not the case with intrauterine retention of fetal bones [1].

In addition to HPE, ultrasonography also plays an important role in the diagnosis of osseous metaplasia. The distinct hyperechogenic pattern with posterior acoustic shadowing observed suggests the presence of osseous tissue in the uterus, a finding that should be validated through hysteroscopic examination [1,8].

Given the rarity of osseous metaplasia, there is no established standard of care. Treatment options may include hysteroscopic removal of bony spicules [7,8,10,13]. Sometimes a hysterectomy is necessary for the definitive treatment of the patient [5]. In the index case, the patient insisted on a hysterectomy as there was no alleviation of symptoms.

## Conclusions

This case report presents a unique instance of osseous metaplasia in a 38-year-old female, detailing the clinical presentation, diagnostic workup, and management of this rare entity. Osseous metaplasia of the endometrium is a rare and intriguing pathological entity. The report highlights the importance of considering uncommon conditions in the differential diagnosis of endometrial abnormalities and underscores the need for further research to better understand the etiology and optimal management strategies for this rare condition.

## References

[1] Rosa-E-Silva JC, Barcelos ID, Navarro PA, Rosa-E-Silva AC, Nogueira AA, Ferriani RA (2009). Osseous metaplasia of the endometrium associated with infertility: a case report and review of the literature. J Med Case Rep.

[2] Umashankar T, Patted S, Handigund R (2010). Endometrial osseous metaplasia: clinicopathological study of a case and literature review. J Hum Reprod Sci.

[3] Patil S, Narchal S, Paricharak D, More S (2013). Endometrial osseous metaplasia: case report with literature review. Ann Med Health Sci Res.

[4] Ramya T, Chitra TV, Poornima C, Anjana B (2013). Endometrial osseous metaplasia: clinicopathological study of a case and literature review. Int J Reprod Contracept Obstet Gynecol.

[5] Magudapathi C, Anathakrishnan R, Kalargala H (2015). Osseous metaplasia of the endometrium: a rare entity. J Obstet Gynaecol India.

[6] Garg D, Bekker G, Akselrod F, Narasimhulu DM (2015). Endometrial osseous metaplasia: an unusual cause of infertility. BMJ Case Rep.

[7] Cayuela E, Perez-Medina T, Vilanova J, Alejo M, Cañadas P (2009). True osseous metaplasia of the endometrium: the bone is not from a fetus. Fertil Steril.

[8] Garzon S, Laganà AS, Carugno J (2021). Osseous metaplasia of the endometrium: a multicenter retrospective study. Eur J Obstet Gynecol Reprod Biol.

[9] Acharya U, Pinion SB, Parkin DE, Hamilton MP (1993). Osseous metaplasia of the endometrium treated by hysteroscopic resection. Br J Obstet Gynaecol.

[10] Bahçeci M, Demirel LC (1996). Osseous metaplasia of the endometrium: a rare cause of infertility and its hysteroscopic management. Hum Reprod.

[11] Shimizu M, Nakayama M (1997). Endometrial ossification in a postmenopausal woman. J Clin Pathol.

[12] Singh P, Kapur K, Singla S, Naz N (2011). Endometrial osseous metaplasia and mature bone formation with extramedullary hematopoiesis. J Hum Reprod Sci.

[13] Venkatesh YS, Stephen SN, Subbaiah M, Badhe BA, Dorairajan G (2021). Malakoplakia of endometrium with osseous metaplasia on evaluation of postmenopausal leukorrhea: a rare case report. J Midlife Health.

[14] Sugino N, Shimamura K, Takiguchi S (1996). Changes in activity of superoxide dismutase in the human endometrium throughout the menstrual cycle and in early pregnancy. Hum Reprod.

[15] Melius FA, Julian TM, Nagel TC (1991). Prolonged retention of intrauterine bones. Obstet Gynecol.

